# Barriers and facilitators for sustainable fall prevention in a Chinese tertiary hospital: perspectives from nurses and managers

**DOI:** 10.3389/fpubh.2026.1939324

**Published:** 2026-10-12

**Authors:** Xiang Chen, Mi Zhao, Zhenhui Tao, Bei Li, Dan Zhu, Jingwen Meng, Mengyun Yang, Ziye Huang, Chenchen Zhao, Chunyue Li, Qiaohong Wang

**Affiliations:** 1 Department of Nursing, Peking University First Hospital, Beijing, China; 2 Department of Hepatobiliary and Pancreatic Surgery, Peking University First Hospital, Beijing, China; 3 Pediatric Department, Peking University First Hospital, Beijing, China; 4 Scientific Research Department, Peking University First Hospital, Beijing, China

**Keywords:** evidence-based practice, fall prevention, falls, hospitals, qualitative research

## Abstract

**Objectives:**

To investigate the facilitators and barriers influencing the implementation of a sustainability program for preventing falls and reducing injury from falls among hospitalized patients, using the Consolidated Framework for Implementation Research (CFIR).

**Methods:**

The sample consisted of 21 nurse managers and key nursing staff from nine high-fall-risk pilot wards at a tertiary hospital in Beijing, China. Under the structural guidance of the CFIR, we collected data through semi-structured interviews with all participants. By conducting a qualitative descriptive analysis of this data, we identified key determinants influencing the implementation of fall prevention measures.

**Results:**

The study identified 23 implementation determinants, comprising 9 barriers, 2 neutral factors, and 12 facilitators, spanning all five domains and 19 of the 39 constructs within the CFIR framework. This study identified three key facilitators: the program’s combination of scientific rigor and practical applicability, the presence of a supportive climate for evidence-based change, and strong institutional leadership backing. Conversely, several key barriers were identified, including insufficient nurse knowledge regarding fall risk screening and multifactorial assessment, challenges in determining rescreening timing, inadequate information systems, a shortage of nurses with evidence-based skills and practical experience, among others.

**Conclusion:**

Multiple barriers and facilitators critically influence the implementation of the inpatient program for preventing falls and reducing injury from falls, and these factors are decisive for its successful execution. The findings establish a foundation for the development of effective implementation strategies to facilitate the integration of evidence into clinical practice, thereby improving fall management and reducing fall-related injuries.

## Introduction

Patient falls represent a significant global public health issue, imposing a substantial burden on healthcare systems (1, 2). Multiple factors contribute to elevated fall risk among hospitalized patients, including advanced age, polypharmacy-related adverse effects, comorbid conditions, and disorientation in unfamiliar clinical settings (3). As reported in the literature, fall rates demonstrate significant disparities, with figures spanning 0.05 to 18.20 per 1,000 patient days, dictated by a combination of national, regional, departmental, and patient-specific variables (4–6). Furthermore, the substantial rate of fall-related injuries presents a significant concern, as evidenced by a nationwide study of 490 tertiary hospitals in China, which reported a median injury rate per fall of 73.68% (5). Falls in hospitals not only lead to physical and psychological harm, poorer health-related quality of life, and extended hospital stays, but also impose greater healthcare costs on the system (7–9). Moreover, the incidence of patient falls and resultant injuries serves as a nursing-sensitive indicator, as it is directly influenced by the scope and quality of nursing care provided (10–12).

Hospital falls arise from a complex interplay of multiple factors, including patient-related characteristics, caregiver influences, environmental conditions, medication effects, and gaps in care delivery processes, all of which may elevate the risk of occurrence (13, 14). Therefore, effective patient safety strategies in clinical settings should encompass the application of risk evaluation instruments, the design of preventive care protocols, and the systematic investigation of contributing factors (15, 16). Falls represent a complex phenomenon involving dynamic multifactorial interactions. According to the World Guidelines for Falls Prevention and Management in Older Adults (17), implementation of multifactorial fall risk assessment and subsequent targeted interventions is strongly recommended. Multiple studies have shown that tailored education for both patients and nursing staff probably reduces fall rates. Additionally, multifactorial interventions have shown a tendency to lower fall rates. In contrast, the evidence supporting other intervention components, such as exercise and medication optimization as a standalone intervention, remains less certain (15, 17, 18). Building on this, research has shown that nurses play a central role in fall-prevention strategies, and that effective nursing practices are likely to reduce the incidence of falls (19–22). These findings are consistent with the emerging emphasis on patient-centered approaches in fall prevention. They not only underscore the central role of nurses in fall prevention but also highlight the importance of multifactorial assessment and intervention, which aligns with the core content of our fall prevention program. That is, nurses are key implementers and stakeholders, and it is necessary to conduct multifactorial fall risk assessments and deliver targeted interventions for patients. In recent years, the concept of patient-centered fall risk awareness has gained increasing traction in the field of fall prevention (23). This approach emphasizes the development of personalized, multifactorial fall prevention plans based on individual risk factors, while incorporating patients’specific needs, preferences, and values. By doing so, it enhances adherence to fall interventions and ensures the practical implementation of fall prevention strategies. However, although existing guidelines recommend comprehensive fall prevention measures and most studied interventions consist of bundled strategies (24–28), they lack individualised approaches for hospitalized patients and fail to reflect the core principles of patient-centered care and active patient engagement.

The “Preventing Falls and Reducing Injury from Falls (fourth edition)” is a Best Practice Guideline (BPG) developed by the Best Practice Spotlight Organizations® (BPSO®), this initiative was started by the Registered Nurses’ Association of Ontario (RNAO) (29). It aims to provide healthcare providers and patients with evidence-based recommendations to standardize and scientize fall prevention strategies and mitigate fall-related injuries. As a BPSO® in collaboration with the RNAO since 2017, our hospital actively promotes the translation and application of best practice guidelines. The “Preventing Falls and Reducing Injury from Falls (fourth edition)” is one such BPG, based on which we developed an evidence-based practice (EBP) for fall prevention. Despite initial success in reducing falls through the implementation of this EBP, its long-term sustainability was challenged. Since 2023, the hospital opened a new campus with another 1,200 beds which became a confluence of factors—including rapid expansion, staffing shortages, and a high proportion of junior nurses—contributed to a resurgence of the in-patient fall rate from 0.14‰ to 0.41‰ between December 2023 and April 2024. Moreover, over 40% of these fall incidents resulted in injury. Driven by these findings and institutional safety objectives, a sustainable BPG implementation program was initiated. This project formulated and implemented a series of change strategies, grounded in the best available evidence, which focused on developing specialty-specific protocols, delivering comprehensive training, performing risk screening and multifactorial assessment, implementing personalized interventions based on each risk factor, and enhancing patient engagement. These fall prevention and injury reduction initiatives were piloted in nine high fall-rate nursing units within a Chinese tertiary hospital.

The Consolidated Framework for Implementation Research (CFIR 1.0) (30), an implementation science framework, enables the assessment of barriers and facilitators within specific implementation contexts. It guides researchers to analyze the factors affecting implementation from multiple dimensions and is among the most widely used theoretical frameworks in the field of implementation research. The CFIR encompasses 39 constructs across five key dimensions: intervention characteristics, outer setting, inner setting, characteristics of individuals, and the implementation process (30), as described in Table 1. These elements interact with one another to collectively influence the implementation and outcomes of an intervention. The CFIR Expert Recommendations for Implementation of Change (ERIC) matching tool aids in choosing theory-based implementation strategies (31, 32).

**Table 1:** CFIR domains and their definitions.

CFIR domain	Definition
Intervention characteristics	Refer to the features of the intervention designed for a specific implementation context. This domain comprises eight constructs: intervention source, evidence strength and quality, relative advantage, adaptability, trialability, complexity, design quality and packaging, and cost.
Outer setting	Refers to the external social, economic, and political context that may influence implementation, and comprises four constructs: patient needs and resources, cosmopolitanism, peer pressure, and external policy and incentives.
Inner setting	Refers to organizational factors that shape the implementation context, including internal culture, structure, and networks. It encompasses 12 constructs: structural characteristics, networks and communication, organizational culture, implementation climate (including tension for change, compatibility, relative priority, organizational incentives and rewards, goals and feedback, and learning climate), and readiness for implementation (including leadership engagement, available resources, and access to knowledge and information)
Characteristics of individuals	Refer to the personal attributes of those involved in the implementation process. This domain comprises four constructs: knowledge and beliefs about the intervention, self-efficacy, individual stage of change, individual identification with the organization, and other personal attributes.
Process	Refers to the methods and strategies employed at both individual and organizational levels to facilitate the adoption and integration of an intervention. Including planning, engaging (opinion leaders, formally appointed internal implementation leaders, champions and external change agents), executing, and reflecting and evaluating

Identifying the facilitators and barriers to this evidence-based practice (EBP) project is fundamental to optimizing its application, ensuring its sustainability, and preserving standards of long-term care (33). Our objective was to explore the determinants of its sustained implementation and derive practical recommendations for overcoming impediments. To this end, the present study employed the Consolidated Framework for Implementation Research (CFIR) to examine the factors influencing its implementation. The identified barriers were then matched with theory-informed implementation strategies using the CFIR-ERIC matching tool.

## Methods

### Design

A qualitative descriptive study was conducted with key stakeholders to explore the barriers to and facilitators of implementing the Preventing Falls and Reducing Injury from Falls program for inpatients. The reporting of this qualitative study was guided by the Consolidated Criteria for Reporting Qualitative Research (COREQ) (34).

### Setting and participants

This study was conducted in nine pilot wards at a tertiary hospital in mainland China, which were selected based on their ranking among the wards with the highest fall incidence rates. These wards comprised six internal medicine units and three surgical units, specifically: Cardiology, Neurology, Hematology, Endocrinology, Nephrology, Geriatrics, Interventional Vascular Surgery, Orthopedics, and Cardiac Surgery. An evidence-based fall prevention program for inpatients had recently been introduced in these wards. Data were collected from September to October 2024. The program was designed to reduce fall rates and improve patient safety, and its implementation provided a relevant context for this research. The study employed purposive sampling to recruit nurse managers and key nursing staff from nine pilot wards participating in the fall prevention program. Inclusion criteria for nurses were: (1) possessing relevant professionally qualified certificates recognized by the state, (2) participating in this evidence-based practice project on fall prevention and have at least 3 years of work experience, and (3) being able to provide signed informed consent. The exclusion Criteria were: (1) rotating nurse residents and nursing interns, and (2) personnel not on duty such as those on maternity or paternity leave.

### Data collection

Data were collected through face-to-face semi-structured interviews conducted by the first authors, trained graduate researchers with extensive clinical experience and formal training in qualitative methods. Individual interviews were conducted with nurse managers and key nursing staff to gather detailed perspectives on the implementation process. The interview guide was formulated in accordance with the CFIR implementation guide and encompassed five key dimensions: intervention characteristics (4 questions), outer setting (2 questions), inner setting (5 questions), individual characteristics (1 question), and process (3 questions). Table 2 provides a complete overview of the interview guide. To develop the interview guide, we referenced the question templates provided on the CFIR official website, and subsequently refined it through pre-interviews with two participants and consultations with qualitative research experts. The interviewer had no supervisory or hierarchical relationship with any of the participants, which helped minimize potential power imbalances. Participants were informed that the interviewer was independent of their clinical performance evaluation and that their responses would remain confidential and anonymized. Nurse managers and key nursing staff from the nine pilot wards were purposively sampled. Initial contact was made through the nursing department, followed by direct invitations to eligible participants via in-person communication. All potential participants received a study information sheet explaining the purpose of the study, the voluntary nature of participation, and the measures taken to protect confidentiality. After the study purpose was explained, all participants signed written informed consent forms and completed a brief demographic questionnaire. Interviews took place in a private, quiet room away from clinical areas to ensure privacy and were audio-recorded with participants’ consent. Before each interview, the interviewer emphasized voluntary participation and assured participants of their right to withdraw at any time without consequences.

**Table 2:** Interview guide.

CFIR construct	Interview questions
Intervention characteristics	1. What advantages do you believe about this program, and what difficulties and suggestions do you have?
	2. In your opinion, what adjustments can be made to this fall prevention project to make it more closely aligned with the reality of the ward?
	3. During the implementation of this intervention program, what difficulties do you anticipate may arise in the future, and what suggestions do you have?
	4. What advantages do you see in the bundled presentation of this project’s content?
Outer setting	5. Do you think this project takes into account the individual needs and preferences of patients?
	6. Do you believe there is any peer pressure associated with this program?
Inner setting	7. During the process of advancing the project, what formal and informal (please provide examples) communications do you anticipate will occur internally in the future?
	8. How do you find the multidisciplinary collaboration channels? What difficulties have you encountered, and do you have any suggestions?
	9. How do you perceive the importance and urgency of this evidence-based fall prevention project?
	10. How do you think this program will be received by everyone in the future?
	11. What kind of support and motivation do you anticipate?
Characteristics of individuals	12. Do you feel that you have sufficient capabilities to implement the intervention program to achieve the implementation goals?
Process	13. Who are the key individuals in the organization to get on board with implementation? Are there other people in the organization likely to champion the intervention?
	14. How about the project plan and quality of carrying out the project?
	15. Is there any feedback and the style of feedback? How often?

### Data analysis

We adopted a qualitative content analysis approach guided by the CFIR 1.0 domains and constructs. Data were collected and analysed concurrently, with NVivo 12 software used to assist data management and analysis. Following the thematic analysis procedures outlined by Braun and Clarke (35), three trained researchers independently reviewed all transcripts and conducted line-by-line coding to identify recurring topics. Initial codes were generated based on the data, then grouped into sub-themes and refined iteratively. In a secondary, interpretive phase, sub-themes were mapped to relevant CFIR constructs using the official CFIR coding manual. Discrepancies were resolved through consensus discussion, with methodological guidance from an experienced qualitative researcher. All authors reviewed and refined the thematic structure. An audit trail was maintained to ensure transparency and trustworthiness.

## Results

Face-to-face semi-structured interviews were conducted with a purposive sample of 21 participants from pilot wards, comprising 9 nurse managers and 12 senior staff nurses. All participants were female, 95 % of the participants held a bachelor’s degree or higher, while 62% had intermediate-level professional titles or higher. The length of the interviews ranged from 40 to 60 min. Participant demographic characteristics are summarized in Table 3. A total of 9 barriers, 2 neutral factor and 12 facilitators were identified in the CFIR domains (See Table 4).

**Table 3:** Demographic characteristics of the participants (*N* = 21).

Participant	*N* = 21
Age (years)	
20–29	4
30–39	15
≥40	2
Education level	
Master’s degree	2
Bachelor’s degree	18
College diploma	1
Years of work	
<5	4
5–10	6
11–20	9
≥21	2
Characteristics	
Nurse manager	9
Key nursing staff	12

**Table 4:** Barriers and facilitators to implementing the fall prevention program.

CFIR domains and constructs	Study results
Intervention characteristics: four barriers and four facilitators	
Evidence strength and quality	One facilitator
Trial ability	One facilitator
Relative advantage	One facilitator
Complexity	Three barriers
Design quality and packaging	One facilitator
Cost	One barrier
Outer setting: one neutral factor and two facilitators	
Patient needs and resources	One facilitator
Cosmopolitanism	One facilitator
Peer pressure	One neutral factor
Inner setting: three barriers, one neutral factor and three facilitators	
Structural characteristics	Two barriers
Culture	One neutral factor
Implementation climate	Two facilitators
Leadership engagement	One facilitator
Relative priority	One barrier
Characteristics of individuals: two barriers and one facilitator	
Knowledge and beliefs about the intervention	One barrier
Self-efficacy	One barrier
Individual identification with organization	One facilitator
Process: two facilitators	
Planning	One facilitator
Reflecting and evaluating	One facilitator

### Theme 1: Intervention characteristics

#### Subtheme 1.1: evidence strength and quality

This fall prevention program is based on evidence-based medicine principles, with supporting evidence derived from high-quality clinical guidelines and evidence summaries. Both the interviewed nurse managers and senior staff nurses expressed strong confidence in the evidence quality and effectiveness of this intervention. Participant 8 said, “This project demonstrates high-quality evidence, providing effective guidance and a clear framework for clinical practice.” Participant 12 reported, “The new program provides more comprehensive and targeted assessment and intervention for fall prevention.”

#### Subtheme 1.2: trial ability

The interviewed nurses and managers generally agreed that the project was clear, specific, and clinically feasible. Participant 18 stated, “This initiative may first undergo pilot implementation in high fall-rate wards, with subsequent hospital-wide dissemination upon protocol maturation.” Participant 1 said, “This project features a clear and well-structured framework, making it suitable for initial pilot implementation in selected wards.”

#### Subtheme 1.3: relative advantage

The participants widely recognized the significant relative advantages of the new fall prevention measures compared to conventional strategies, primarily reflected in their detailed protocols, enhanced professionalism and specificity, which enable effective identification and intervention for high-risk patients with diverse fall etiologies. Participant 18 stated, “The new version of the intervention protocol in this fall prevention program is both detailed and comprehensive in content.”

#### Subtheme 1.4: complexity

Interview participants widely agreed that the implementation process of the program was complex and time-consuming. Additionally, the re-assessment timing for the new fall risk assessment tool was unclear, making it difficult for clinical nurses to apply consistently and leading to potential missed evaluations. Furthermore, the fall prevention protocol lacked clear definitions and boundaries regarding “patient involvement,” and the knowledge base for fall prevention was too general, failing to reflect specialty-specific considerations. Participant 2 said, “The reassessment criteria in the protocol are too flexible—some do not apply to our ward, while others lack specialty-specific considerations, which may lead to missed evaluations.” Participant 5 reported, “The fall prevention knowledge base provided in the protocol lacks some specialty-specific content relevant to our ward—content that is frequently encountered in our patients with fall risks.”

#### Subtheme 1.5: design quality and packaging

The new fall prevention program not only provides a clear operational framework and diversified health education resources, but also features seamless integration with the information system. It automatically extracts patients’ basic data to generate corresponding nursing issues, thereby helping nurses master the application method more effectively and reduce decision-making costs. Participant 10 said, “The new fall risk assessment tool features automated patient data retrieval through the information system, simplifying the evaluation process. Furthermore, the system automatically generates corresponding nursing diagnoses and intervention recommendations based on multifactorial assessment results.”

#### Subtheme 1.6: cost

The fall prevention program involves multiple stakeholders and incurs significant human resource and time costs during implementation. During the initial phase, nursing staff require substantial time for training and adaptation to the new assessment protocols due to unfamiliarity with the revised workflow. Participant 7 reported, “The updated fall risk assessment tool is overly detailed and time-consuming to complete. Given our already heavy workload, we sometimes lack the patience to carefully evaluate every single item.”

### Theme 2: Outer setting

#### Subtheme 2.1: patient needs and resources

The participants widely acknowledged that the program thoroughly addressed both the needs and preferences of patients and their caregivers, incorporating “patient engagement in fall prevention” as a key audit criteria. Furthermore, assessments of patients’ knowledge, attitudes, and practices (KAP) regarding fall prevention were conducted before and after program implementation to facilitate more precisely tailored interventions. Participant 5 said, “Through preliminary questionnaire surveys, we have ascertained ward patients’ awareness level and knowledge base regarding fall prevention.” Despite thorough consideration of both patients’ and caregivers’ preferences during program implementation, the actual execution outcomes remained suboptimal. Participant 16 stated, “The new protocol places greater emphasis on patient and caregiver participation and feedback; however, our ward still encountered implementation challenges.”

#### Subtheme 2.2: cosmopolitanism

Enhancing staff’s cosmopolitanism increases organizational readiness for rapid change implementation. Furthermore, sufficient internal collaboration within the organization is also a key factor in facilitating project implementation. Participant 12 mentioned, “Our hospital’s close collaboration with external resources such as the Chinese Nursing Association, Peking University School of Nursing, and JBI was crucial for the successful implementation of the hospital-based evidence translation project.”

#### Subtheme 2.3: peer pressure

Some participants learned that other hospitals also have comprehensive fall prevention programs, though our program emphasizes multifactorial assessment, thoroughly considers patients’ fall-related factors, and provides corresponding targeted nursing interventions. Participant 6 said, “Some hospitals with earlier-developed health information systems have implemented clinical decision support system concepts through nursing electronic medical records (NEMRs), significantly reducing nurses’ decision-making costs in fall risk screening, assessment, and intervention planning processes.”

### Theme 3: Inner setting

#### Subtheme 3.1: structural characteristics

Multiple participants noted the ward’s shortage of nursing staff trained in systematic evidence-based knowledge, resulting in limited support when developing specialized fall prevention protocols. They recommended expanding the coverage of hospital-level evidence-based nursing training programs to enhance clinical nurses’ evidence-based knowledge and practice competencies.

#### Subtheme 3.2: culture

Participants recognized that fall prevention is essential for inpatient safety and that the program is suitable for implementation across all hospital wards without cultural barriers. However, clinicians and physiotherapists in this multidisciplinary team placed greater emphasis on patient treatment outcomes, demonstrating insufficient attention to and engagement with fall prevention initiatives.

#### Subtheme 3.3: implementation climate

The majority of participants acknowledged strong leadership support for the evidence-based practice initiative, noting a favorable implementation climate and high readiness for change among nursing staff, who found the fall prevention protocol highly applicable to clinical practice. Furthermore, both ward operations and patients themselves benefited from the program, while clinical nurses reported enhanced professional value and sense of accomplishment.

#### Subtheme 3.4: leadership engagement

Participants identified leadership engagement as a significant influencing factor. The majority of participants perceived that the strong commitment from the nursing department leadership, particularly the deputy director’s continuous involvement and chairing of the initiative, positively influenced the implementation of the fall prevention program. Participant 7 mentioned, “Falls represent a critical risk factor compromising inpatient safety and a core quality indicator in clinical nursing practice. This fall prevention program initiated by the Nursing Department addresses a long-standing priority for ward managers.”

#### Subtheme 3.5: relative priority

The majority of participants perceived falls prevention programs as facing competitive disadvantages in resource allocation—despite being a priority patient safety issue—due to ward-level considerations of cost–benefit ratios. Participant 9 stated, “We are fully receptive to adopting these interventions, provided they do not incur additional costs or increase nursing workload.”

### Theme 4: Characteristics of individuals

#### Subtheme 4.1: knowledge and beliefs about the intervention

Participants acknowledged the clinical utility of the fall prevention intervention, yet recognized that some risk factors were unavoidable, and nurses expressed concerns about disproportionate effort-to-outcome ratios. Participant 15 said, “I remain skeptical that this program can significantly reduce fall incidence rates, given that patient falls stem from multifactorial causes, most of which are non-modifiable risk factors.”

#### Subtheme 4.2: self-efficacy

Participants generally exhibited low self-efficacy regarding program implementation, primarily attributable to time constraints during rollout, staffing shortages following the new campus opening, and increased workload demands. Participant 12 said, “As a newly established ward in our hospital’s expansion campus, with a relatively inexperienced nursing team still working to standardize our routines, I have serious concerns about our ability to implement this project effectively within such a tight timeframe.

#### Subtheme 4.3: individual identification with organization

While some concerns existed regarding project implementation, the majority of participants expressed confidence in the research team and demonstrated willingness to actively engage in the fall prevention initiative.

### Theme 5: Process

#### Subtheme 5.1: planning

The project was executed following a clear implementation plan. The intervention protocol underwent a rigorous three-month pre-implementation development phase and was revised after a group meeting with 9 pilot wards. The project was developed based on the JBI Evidence Implementation Framework, integrating evidence from JBI and international clinical guidelines. It systematically established an evidence-based practice program through five key components: (1) protocol development, (2) competency-based staff education, (3) screening and multifactorial assessment, (4) individualized intervention protocols, and (5) patient-centered engagement strategies. Additionally, the fall risk assessment and prevention module were embedded into the hospital information system to enhance nurses’ acceptance of the program and clinical applicability.

#### Subtheme 5.2: reflecting and evaluating

Participants generally agreed that the monthly online meetings for the fall prevention program, along with the quarterly Continuous Quality Improvement (CQI) meetings, contributed to enhanced work efficiency and project implementation. Crossover audit among pilot wards helped create constructive competitive pressure and implementation momentum. Concerning the reflection and evaluation process, most participants acknowledged that implementing this project may initially increase nursing staff workload, complicate fall risk assessment procedures, and potentially elicit resistance from some nurses. However, nursing professionals generally agreed that reducing the incidence of fall-related adverse events holds greater value than the added workload. With ongoing updates and maintenance of the information system, the fall assessment process is expected to become more efficient and intelligent.

### Implementation strategies identified using the CFIR-ERIC matching tool

Based on the barriers identified through the semi-structured interviews (i.e., complexity, cost, structural characteristics, relative priority, knowledge and beliefs about the intervention, and self-efficacy), the research team systematically input all identified barriers into the CFIR-ERIC Matching Tool (31, 32), generating a standardized set of strategy recommendations. The most frequently recommended implementation strategies across CFIR constructs included: (1) identifying and preparing individual and group-level champions to support inpatient fall prevention program implementation, (2) assessing organizational readiness while identifying context-specific barriers and facilitators, and (3) modifying incentive structures and resource allocation mechanisms to enhance implementation. Details are shown in Table 5.

**Table 5:** Top 10 implementation strategies for the fall prevention program in inpatients.

ERIC strategy	Number of relevant CFIR constructs	Relevant CFIR constructs
1. Identify and prepare champions	4	Complexity, structural characteristics, knowledge and beliefs about the intervention, self-efficacy
2. Assess for readiness and identify barriers and facilitators	4	Complexity, structural characteristics, relative priority、knowledge and beliefs about the intervention
3. Alter incentive/allowance structures	2	Cost, relative priority
4. Promote adaptability	2	Complexity, structural characteristics
5. Capture and share local knowledge	3	Complexity, structural characteristics, knowledge and beliefs about the intervention
6. Conduct cyclical small tests of change	3	Complexity、Structural Characteristics, self-efficacy
7. Create a learning collaborative	2	Complexity, self-efficacy
8. Conduct educational meetings	1	Knowledge and beliefs about the intervention
9. Develop a formal implementation blueprint	1	Complexity
10. Access new funding	1	Cost

## Discussion

To our knowledge, this is the first study in China to identify the facilitators and barriers to achieving sustainability of an RNAO BPG for hospitalized patients. In this qualitative study, we used the CFIR to explore the perspectives of nurses as key stakeholders on the sustainability of the evidence-based fall prevention program. Semi-structured interviews were conducted with 21 nurses, including both nurse managers and key clinical staff, from wards with high fall incidence rates. All participants were purposively sampled from high-risk wards within the hospital, ensuring strong representativeness of the sample. In total, based on the CFIR, 23 implementation determinants including two neutral influencing factors, 9 barriers and 12 facilitators, were identified in the study. Rather than referring to a passive continuation of a prior initiative, sustainability in our study reflects an active process of renewal and optimization. We consider this a new implementation effort built upon previous experience. Accordingly, we adopted the CFIR framework to identify barriers and facilitators during this early phase of the renewed implementation, with the aim of generating insights that can support long-term maintenance in the future.

This study identified 12 facilitators, which primarily encompassed the following aspects. Firstly, the program demonstrated both scientific rigor and practical applicability. Developed based on a synthesis of fall prevention guidelines and high-quality evidence (29, 36–39), it incorporated key components such as fall risk screening, multifactorial risk assessment, and personalized intervention strategies. Moreover, the program was specifically tailored to the characteristics of the Chinese healthcare context, thereby enhancing its feasibility for clinical implementation by healthcare professionals. In this study, the fall risk screening and multifactorial assessment components were comprehensive and well-validated, encompassing a wide range of potential risk factors associated with in-hospital falls. This enables nurses to accurately identify high-risk patients and implement targeted preventive measures. Secondly, the hospital in which this fall prevention program was implemented had prior experience with evidence-based nursing initiatives, providing a foundational research infrastructure and a supportive climate for change. Healthcare professionals generally held positive attitudes toward the implementation of this evidence-based program, which further facilitated its successful integration and effective execution in clinical practice. Finally, strong support was provided by hospital leadership for the implementation of the program, including the provision of adequate resources such as funding, time, and personnel. In addition, the hospital’s information technology department collaborated in developing a corresponding digital assessment system, offering technical support to ensure that the fall-related assessment forms were user-friendly and aligned with routine nursing practices and cultural norms.

Barriers to intervention characteristics mainly focused on complexity and costs. In the context of fall risk screening and assessment, our hospital previously conducted risk screening only for inpatients at fixed weekly intervals, which did not incorporate nurses’ critical thinking regarding changes in patients’ conditions. In contrast, the updated fall prevention program—developed based on clinical guidelines—includes fall risk screening for all inpatients, followed by a more detailed multifactorial risk assessment (e.g., toileting, nutrition, medication) for those identified as high-risk, with reassessment triggered by changes in clinical status. Healthcare professionals’ motivation to adhere to clinical practice guidelines is critically determined by their knowledge, competencies, and attitudes (40). In this study, although participants reported that the new program required greater time investment and that the more flexible reassessment schedule could lead to missed evaluations—particularly among novice nurses—they widely agreed that the tool enabled more comprehensive and accurate identification of fall risk factors. Despite the presence of positive attitudes among some nurses, the implementation of the program inevitably led to a short-term increase in nursing workload, demanding greater time investment in fall prevention activities. Similarly, Gibson et al. (41) found that issues including staff shortage, heavy workload and limited time frequently hindered nurses from executing standard fall risk policies. According to the 2018 National Statistics of Nurse Resources, China’s number of nurses per 1,000 population (*n* = 2.7) was lower than the global average (*n* = 3.4) and substantially lower than the figures reported in high-income Western countries (42). Therefore, staff shortages and excessive workloads pose a more significant challenge for nurses in China. It is therefore critical to address the compatibility between the fall prevention program and existing workflows and to leverage the facilitators identified in this study, such as leadership engagement and support, a climate for change, and strong support from the hospital informatics department. Such solutions can help reduce the workload and time burden associated with new interventions for nursing staff, thereby increasing the likelihood of successfully implementing evidence-based practices in busy clinical settings. Furthermore, advancing the level of informatization in hospital nursing systems by transforming complex assessment processes into clear electronic pathways, enabling automatic retrieval of foundational data, providing structured guidance, and implementing automated reassessment reminders serves as a powerful tool for promoting the implementation of nursing standards and enabling continuous quality improvement. Research indicates that the integration of fall risk assessment tools and related information technology into healthcare professionals’ daily workflows is critically contingent upon the tools’ ease of use and their compatibility with existing work patterns (43). Therefore, enhancing collaboration with hospital information technology personnel to improve the usability and intelligence of electronic fall screening and assessment forms is a critical measure for facilitating evidence-based nursing practice in fall prevention.

Participants perceived the lack of specific policies for the fall prevention program and the absence of corresponding incentive mechanisms as a significant barrier to its implementation. In the Chinese context, this policy gap may lead healthcare professionals to perceive the implementation of new interventions as potentially risky. Well-defined policies can provide clear standards and guidance for implementing fall prevention measures, while incentive mechanisms can encourage healthcare professionals to adhere to these protocols and prioritize patient fall risk management. Therefore, both tangible and intangible incentive mechanisms should be implemented, such as providing expanded opportunities for off-site training to nursing staff in pilot wards, or enhancing motivation through awards and public recognition programs.

Deficiencies in the development of a multidisciplinary team for evidence-based fall prevention practice, coupled with an insufficient reserve of local champions, were identified as significant barriers to implementation. Establishing multidisciplinary collaboration and clarifying role responsibilities are crucial for ensuring the reliable transmission of key information within the team, thereby contributing significantly to the successful implementation of fall prevention programs (44). In this study, respondents indicated that physicians, physiotherapists, and pharmacists tended to prioritize patient treatment outcomes, while paying insufficient attention to fall risk management. Biological factors, however, represent one of the most significant contributors to patient falls, closely associated with disease characteristics, physiological decline, and medication side effects. Therefore, it is imperative to enhance the emphasis on fall risk management within multidisciplinary collaboration by integrating fall prevention into overall treatment goals, thereby balancing patient safety and therapeutic efficacy. Furthermore, some participants noted that nurses lacked adequate evidence-based knowledge and skills, resulting in fall prevention protocols that either lacked specialty-specific preventive content or were not supported by high-level evidence. This limitation impeded the development of precise and effective fall prevention strategies tailored to the characteristics of patients in different departments, ultimately compromising the clinical applicability and implementation effectiveness of the interventions. Establishing a researcher-practitioner partnership to promote evidence-based development of specialty-specific fall prevention strategies could help address this barrier.

## Limitations

This study has several limitations. First, it was conducted within a single tertiary hospital in China; consequently, the facilitators identified may be context-specific and might not be readily generalizable to other settings. Second, our sample included only nursing managers and core nursing staff, as they were the primary implementers and key stakeholders throughout the program. Future research should consider incorporating multiple stakeholder perspectives, such as those of physicians, physiotherapists, pharmacists, patients, and family caregivers, to obtain a more comprehensive understanding of implementation dynamics.

## Conclusion

Although falls have emerged as a global public health concern with numerous guidelines and high-level evidence available for prevention, a significant gap remains between clinical practice and best evidence, leading to suboptimal outcomes in inpatient fall prevention and post-fall injury reduction management. Using the CFIR, this study identified barriers and facilitators to evidence-based practice for inpatient fall prevention and post-fall injury reduction in the Chinese context. The findings provide a foundation for developing effective implementation strategies to integrate evidence into the management of inpatient fall prevention and mitigation of fall-related injuries.

## Data Availability

The data that support the findings of this study are available upon request from the corresponding author. The data are not publicly available due to privacy and ethical restrictions.
